# Plasma Concentrations and Dietary Intakes of Choline and Betaine in Association With Atrial Fibrillation Risk: Results From 3 Prospective Cohorts With Different Health Profiles

**DOI:** 10.1161/JAHA.117.008190

**Published:** 2018-04-12

**Authors:** Hui Zuo, Gard F. T. Svingen, Grethe S. Tell, Per M. Ueland, Stein E. Vollset, Eva R. Pedersen, Arve Ulvik, Klaus Meyer, Jan E. Nordrehaug, Dennis W. T. Nilsen, Kaare H. Bønaa, Ottar Nygård

**Affiliations:** ^1^ Department of Global Public Health and Primary Care University of Bergen Norway; ^2^ Department of Clinical Science University of Bergen Norway; ^3^ Department of Heart Disease Haukeland University Hospital Bergen Norway; ^4^ Laboratory of Clinical Biochemistry Haukeland University Hospital Bergen Norway; ^5^ Division of Mental and Physical Health Department of Noncommunicable Diseases Norwegian Institute of Public Health Bergen Norway; ^6^ Centre for Burden of Disease Norwegian Institute of Public Health Bergen Norway; ^7^ Bevital A/S Bergen Norway; ^8^ Department of Cardiology Stavanger University Hospital Stavanger Norway; ^9^ Department of Public Health and Nursing Norwegian University of Science and Technology Trondheim Norway; ^10^ Department of Community Medicine The Arctic University of Norway Tromsø Norway; ^11^ Clinic for Heart Disease St. Olav's University Hospital Trondheim Norway

**Keywords:** atrial fibrillation, betaine, choline, cohort studies, the CVDNOR project, Atrial Fibrillation, Metabolism, Epidemiology

## Abstract

**Background:**

Although choline metabolism has been associated with atherosclerotic heart disease, less research attention has been paid to the associations of choline and its oxidative metabolite betaine with cardiac arrhythmias.

**Methods and Results:**

We evaluated associations of plasma concentrations and dietary intakes of choline and betaine with long‐term atrial fibrillation (AF) risk in a community‐based cohort, HUSK ([the Hordaland Health Study] n=6949), and validated the findings in 2 patient cohorts: the Western Norway Coronary Angiography Cohort (n=4164) and the NORVIT (Norwegian B‐Vitamin) Trial (n=3733). Information on AF was obtained from the CVDNOR (Cardiovascular Disease in Norway) project. In HUSK, WECAC (Western Norway Coronary Angiography Cohort), and NORVIT, 552, 411, and 663 AF cases were identified during a median follow‐up time of 10.9, 7.3, and, 8.7 years, respectively. Plasma concentrations of choline and betaine were significantly positively associated with later AF risk after multivariable adjustments in HUSK. Such associations were independently replicated in the 2 external prospective patient cohorts. The pooled hazard ratio was 1.13 (95% confidence interval 1.08‐1.19, *P*<0.001) and 1.16 (95% confidence interval 1.10‐1.22, *P*<0.001) per SD increment for log‐transformed choline and betaine, respectively. Moreover, dietary intake of choline was marginally associated with AF risk (pooled hazard ratio 1.29, 95% confidence interval 1.01‐1.66, fifth versus first quintile), whereas no significant association was observed between dietary betaine and AF risk.

**Conclusions:**

Our findings indicate that plasma concentrations as well as dietary intake of choline, but not betaine, are associated with subsequent risk of AF, suggesting a potential role of choline metabolism in the pathogenesis of AF.

**Clinical Trial Registration:**

URL: https://www.clinicaltrials.gov.Unique identifier: NCT00671346.

**Clinical Perspective**

**What Is New?**

- Higher plasma concentrations of choline and betaine were associated with an increased risk of atrial fibrillation.
- Dietary intake of total choline was marginally associated with atrial fibrillation risk and betaine intake not associated.

**What Are the Clinical Implications?**

- Our findings suggest a potential role of choline metabolism in atrial fibrillation pathogenesis and may help to target individuals predisposed to develop atrial fibrillation for potential interventions.

Atrial fibrillation (AF) is the most common clinically relevant cardiac arrhythmia linked to excess morbidity and mortality.^1^ It has emerged as a global epidemic due to progressively increasing incidence and prevalence worldwide, with developed countries having a greater burden.^2^ This underscores the importance of disease prevention by identifying novel risk markers because traditional AF risk factors such as higher age, male sex, obesity, diabetes mellitus, and hypertension do not fully explain the variance in AF risk.^3^

Choline is an essential nutrient but can also be derived from endogenous biosynthesis.^4^ The total choline in foods includes free choline in addition to choline esters.^5^ In the mitochondria, choline is oxidized to betaine, which serves as an intracellular osmolyte and methyl donor in the folate‐independent remethylation of homocysteine to methionine, catalyzed by betaine‐homocysteine methyltransferase, a reaction producing dimethylglycine.^4^, ^6^ In addition to de novo synthesis primarily in liver and kidney, betaine is also obtained from dietary sources.^4^

No associations of dietary choline and betaine with cardiovascular disease (CVD) risk have been reported,^7^, ^8^, ^9^ whereas circulating choline and its metabolites have been associated with lifestyle diseases including atherosclerotic cardiovascular disease.^4^, ^6^, ^10^, ^11^ However, less attention has been paid to the possible associations between choline metabolites and cardiac arrhythmias, although a recent metabolomics study suggested no significant association between plasma betaine and incident AF in the population‐based Framingham Study.^12^

The current study therefore investigated whether plasma choline and betaine, as well as their dietary intakes, were associated with subsequent AF over long‐term follow‐up. The primary cohort consisted of participants from the general population, and the findings were sought replicated in 2 external cohorts: patients with suspected stable angina pectoris and patients hospitalized with acute myocardial infarction.

## Methods

The data, analytic methods, and study materials will not be made available to other researchers for purposes of reproducing the results or replicating the procedure.

### Study Population

The community‐based HUSK (Hordaland Health Study) has been described in detail elsewhere^13^, ^14^ (http://husk.b.uib.no). The present study cohort was confined to 7050 men and women who were born during 1925‐1927 or 1950‐1951 and participated in the baseline examinations in the period of April 1998 to June 1999. Of the 7050 participants, we excluded 65 who were diagnosed with AF before enrollment as well as participants with missing data on plasma biomarkers (n=36). A total of 6949 participants (3072 men and 3877 women) were included in the final analyses.

The 2 patient cohorts, WECAC (the Western Norway Coronary Angiography Cohort) and NORVIT (the Norwegian Vitamin Trial), have been described in more detail previously.^11^ Briefly, WECAC consisted of 5209 patients undergoing coronary angiography during 2000‐2004. Of these, 4164 patients with suspected stable angina pectoris were selected for the current study. About two‐thirds of these patients were included in WENBIT (the Western Norway B‐vitamin Intervention Trial), a randomized clinical trial investigating the effect of B‐vitamin treatment on CVD and mortality.^15^ NORVIT was a randomized clinical trial including 3749 patients hospitalized with acute myocardial infarction in the period 1998‐2002 and randomized to identical treatment protocols as in WENBIT.^16^ In WECAC, we excluded patients with a history of previous AF from the analyses, whereas we did not have information on previous AF among patients in NORVIT.

The study was performed according to the Declaration of Helsinki, and all participants provided written informed consent. The study protocol was approved by the Regional Committee for Medical and Health Research Ethics (REK 2009/825 and 2010/1880).

### Biosampling and Biochemical Analyses

In HUSK, nonfasting blood samples were collected at baseline. Time since last meal was recorded. In WECAC, blood samples were drawn 1 to 3 days before or immediately after the coronary angiography procedure, and 28.3% of patients were fasting. In NORVIT, blood samples were drawn within 1 week after the index acute myocardial infarction, with no information on fasting status.

In HUSK, aliquots of serum and plasma were frozen at −80°C until later analyses. In WECAC and NORVIT, routine laboratory measurements were carried out on fresh blood samples at each recruiting hospital, whereas blood samples for specific analyses were shipped to the core biobank at Haukeland University Hospital for long‐term storage at −80°C. Study‐specific analyses for all the 3 cohorts were carried out at the Bevital A/S Laboratory, Bergen, Norway (http://www.bevital.no) on thawed samples by personnel blinded to the study outcomes.

Plasma choline and betaine were measured by liquid chromatography‐tandem mass spectrometry.^17^, ^18^ Serum folate was measured by a microbiological assay.^19^ Serum creatinine was measured colorimetrically using the alkaline picrate method with reagents from Roche (Basle, Switzerland).^13^ Plasma high‐sensitivity C‐reactive protein (CRP) was determined by an immuno‐MALDI‐MS method.^20^ Within‐day coefficients of variation for choline and betaine were 5.4% to 7.6%, and between‐day coefficients of variation were 3.8% to 9.5%.^17^ The limit of detection was 0.50 μmol/L for both choline and betaine and 0.10 mg/L for CRP. The limit of quantitation was 1.0 μmol/L for both choline and betaine and 0.16 mg/L for CRP.

### Follow‐Up and Outcome Ascertainment

Cohort participants were followed from baseline to the date of AF diagnosis, death, emigration, or through December 31, 2009 (the end of follow‐up), whichever came first. Information on hospitalization with an AF diagnosis was obtained via linkage to hospital discharge diagnosis data obtained through the CVDNOR (Cardiovascular Disease in Norway) project (https://cvdnor.b.uib.no).^21^, ^22^, ^23^ The primary outcome was hospitalization or death attributed to AF (Code 427.3 from *International Classification of Diseases, Ninth Revision* [*ICD‐9*], I48 from *ICD‐10*). Information on death was collected from the Cause of Death Registry at Statistics Norway. A unique 11‐digit personal identifier was used to link baseline variables with study end points.

### Dietary Intake Assessment

Dietary information in HUSK and WECAC was collected with the use of a validated self‐administered food frequency questionnaire.^24^, ^25^ The food frequency questionnaire included 169 food items and offered alternatives for frequency, number of units consumed, and portion sizes to capture the habitual dietary information during the past year. Information on the use of supplements also was obtained from the food frequency questionnaire and included in the calculations. A software system (KBS software, version 3.2; University of Oslo, Norway) was developed to calculate energy and nutrient intakes. Total choline and betaine intakes were estimated according to the US Department of Agriculture's choline database.^5^

### Other Baseline Factors

Smoking status was based on self‐reported smoking status and corrected by plasma cotinine (ie, self‐reported nonsmokers with plasma cotinine concentrations ≥85 nmol/L were reclassified as current smokers).^26^ Blood pressure was measured and body mass index (BMI) calculated based on measured height and weight (kg/m^2^). Calculation of estimated glomerular filtration rate was based on serum creatinine levels using the Chronic Kidney Disease Epidemiology Collaboration equation.^27^

### Statistical Analyses

Continuous and categorical variables are reported as medians (interquartile ranges) and counts (%), respectively. Differences between AF cases and noncases were compared using chi‐squared tests for categorical and Wilcoxon‐Mann‐Whitney tests for continuous variables. Log‐transformation was applied to all plasma biomarkers to normalize their distributions. Spearman partial correlation coefficients were calculated to examine relations between dietary and plasma biomarkers after control for energy intake.

Cox proportional hazards regression was used to calculate hazard ratios (HRs) and 95% confidence intervals (CIs) per 1‐SD increment of log‐transformed plasma concentrations of choline and betaine. In the minimally adjusted models we adjusted for sex and age (continuous). In the multivariable adjusted models we additionally adjusted for a priori selected AF risk factors including BMI (continuous), diabetes mellitus (yes/no), hypertension (yes/no), and smoking (yes/no). An inverse variance‐weighted, fixed‐effect meta‐analysis was used to combine the results across the 3 cohorts because no significant between‐study heterogeneity was found. In HUSK, we also calculated the associations after additional adjustment for estimated glomerular filtration rate (continuous), fasting status (yes/no), education level (≤10, 11‐13, ≥14 years), serum folate (continuous), and plasma CRP (continuous) in the models. In order to address the potential effect of atherosclerotic heart disease on the association between choline/betaine and AF risk, we performed analyses by additional adjustment for CVD/coronary heart disease at baseline in HUSK and WECAC. Potential nonlinear associations between choline/betaine and risk of AF were visualized by smoothed splines from generalized additive models.

To assess potential improvement in model discrimination and reclassification, we calculated the integrated discrimination index, and the continuous net reclassification index (>0) in logistic regression models containing the same covariates as the multivariable Cox model, with and without plasma choline/betaine.^28^, ^29^

Regarding the estimation of associations between dietary intakes of choline and betaine with AF risk, subjects with extreme values of total energy intake (ie, <1st percentile or >99th percentile) for each sex were excluded. Choline and betaine intakes were adjusted for total energy intake by using the residual method.^30^ The consumptions of choline and betaine were categorized into quintiles in each cohort. Multivariable models were adjusted for sex, age (continuous), BMI (continuous), diabetes mellitus (yes/no), hypertension (yes/no), smoking (yes/no), and energy intake (continuous). The results were also pooled by an inverse variance‐weighted, fixed‐effect meta‐analysis.

Statistical analyses were conducted with the SAS statistical package (Version 9.4; SAS Institute, Inc, Cary, NC), SPSS for Windows (Version 23.0, IBM, Armonk, NY), and R (R Core Team 2016, Vienna, Austria, version 3.3.1, http://www.r-project.org). All tests were 2‐sided, and *P*<0.05 was considered statistically significant.

## Results

### Population Characteristics at Baseline

In the 3 cohorts, those who developed AF during follow‐up were in general older at baseline (*P*<0.001), had lower estimated glomerular filtration rate, more frequently had hypertension and diabetes mellitus, and were less likely to be current smokers. Moreover, participants who subsequently developed AF had consistently higher baseline concentrations of plasma choline and betaine (Table 1). In HUSK, those who developed AF had slightly higher energy‐adjusted intake of choline than those who did not. No differences were seen for betaine intake in HUSK or for choline or betaine intake in the WECAC (Table 1).

**Table 1:** Baseline Characteristics of the Study Participants in HUSK, WECAC, and NORVIT

Characteristic	All	AF	No AF	*P* Value^a^
HUSK				
Participants, n	6949	552	6397	
Age, y	48 (47‐72)	72 (71‐73)	48 (47‐72)	<0.001
Men, %	44.2	58.0	42.0	<0.001
BMI, kg/m^2^	25.4 (23.1‐27.9)	25.9 (23.5‐28.8)	25.3 (23.1‐27.8)	<0.001
Current smoking, %	28.6	25.0	28.9	0.05
eGFR, mL/min per 1.73 m^2^	80 (69‐90)	72 (64‐81)	81 (70‐91)	<0.001
Hypertension, %	42.4	63.2	40.6	<0.001
Diabetes mellitus, %	3.8	7.8	3.4	<0.001
CVD, %	9.9	23.4	8.8	<0.001
Plasma/serum biomarkers				
Choline, μmol/L	9.61 (8.28‐11.1)	10.2 (8.82‐12.1)	9.55 (8.23‐11.0)	<0.001
Betaine, μmol/L	38.1 (31.0‐45.9)	41.4 (34.1‐49.2)	37.9 (30.7‐45.6)	<0.001
Folate, nmol/L	6.68 (5.08‐9.04)	6.48 (4.92‐8.91)	6.70 (5.11‐9.05)	0.20
CRP, mg/L	1.56 (0.68‐3.59)	2.29 (1.10‐5.12)	1.50 (0.66‐3.48)	<0.001
Dietary factors				
Energy, kJ/day	7898 (6328‐9780)	7517 (6002‐9184)	7829 (6347‐9842)	<0.001
Total choline intake, mg/day^b^	259.5 (228.0‐294.4)	265.9 (232.1‐301.3)	258.9 (227.8‐293.6)	0.011
Betaine intake, mg/day^b^	130.7 (112.6‐151.3)	131.3 (113.0‐151.8)	130.6 (112.5‐151.3)	0.88
WECAC				
Participants, n	3809	411	3398	
Age, y	61 (54‐69)	68 (61‐74)	60 (54‐68)	<0.001
Men, %	71.7	75.9	71.1	0.043
BMI, kg/m^2^	26.3 (24.2‐28.9)	26.2 (24.2‐29.2)	26.3 (24.2‐28.9)	0.95
Current smoking, %	32.0	26.3	32.7	0.009
eGFR, mL/min per 1.73 m^2^	91 (79‐100)	85 (70‐94)	92 (80‐100)	<0.001
Hypertension, %	46.0	56.9	44.7	<0.001
Diabetes mellitus, %	11.5	15.8	11.0	0.004
CHD, %	45.3	48.9	44.9	0.12
Plasma biomarkers				
Choline, μmol/L	9.62 (8.21‐11.4)	10.30 (8.68‐12.4)	9.55 (8.15‐11.3)	<0.001
Betaine, μmol/L	38.9 (31.8‐47.3)	39.9 (32.9‐51.2)	38.7 (31.7‐46.9)	0.002
Dietary factors				
Energy, kJ/day	8465 (6860‐10 387)	8121 (6557‐9830)	8520 (6907‐10 470)	0.032
Total choline intake, mg/day^b^	247.0 (218.0‐280.9)	248.3 (222.2‐284.0)	246.9 (217.6‐280.5)	0.30
Betaine intake, mg/day^b^	137.8 (118.6‐159.3)	139.8 (123.4‐159.1)	137.6 (117.4‐159.3)	0.24
NORVIT				
Participants, n	3733	663	3070	
Age, y	63 (54‐73)	70 (63‐76)	61 (53‐71)	<0.001
Men, %	74.0	70.8	74.7	0.037
BMI, kg/m^2^	25 (23‐28)	26 (24‐28)	25 (23‐28)	0.06
Current smoking, %	67.7	58.6	69.7	<0.001
eGFR, mL/min per 1.73 m^2^	76 (64‐89)	71 (59‐85)	77 (65‐90)	<0.001
Hypertension, %	29.0	39.2	26.7	<0.001
Diabetes mellitus, %	9.7	12.5	9.1	0.008
Plasma biomarkers				
Choline, μmol/L	10.3 (8.68‐12.2)	11.1 (9.10‐12.9)	10.2 (8.61‐12.0)	<0.001
Betaine, μmol/L	33.1 (27.3‐39.9)	34.0 (28.4‐41.2)	32.9 (27.1‐39.7)	0.002

Values are given as medians (interquartile ranges) or percentages. AF indicates atrial fibrillation; BMI, body mass index; CHD, coronary heart disease; CRP, C‐reactive protein; CVD, cardiovascular disease; eGFR, estimated glomerular filtration rate; HUSK, Hordaland Health Study; NORVIT, Norwegian B‐Vitamin Trial; WECAC, Western Norway Coronary Angiography Cohort.

a Difference between the 2 groups.

b Adjusted for total energy intake. Data analyses were confined to those participants with dietary data (n=5950 in HUSK and 1899 in WECAC).

### Prospective Associations of Plasma Choline and Betaine With Subsequent AF Risk

In HUSK, WECAC, and NORVIT, 552, 411, and 663 AF cases were identified during a median follow‐up time of 10.9, 7.3, and 8.7 years, respectively. Higher plasma concentrations of choline and betaine were associated with an increased risk of developing AF in unadjusted models and in models adjusted for sex, age, BMI, diabetes mellitus, hypertension, and smoking (Table 2). In HUSK, the HR was 1.45 (95% CI 1.33‐1.57, *P*<0.001, log‐transformed, per SD increment) for choline and 1.37 (95% CI 1.26‐1.49, *P*<0.001) for betaine in unadjusted models. These associations remained significant in sex‐ and age‐adjusted models and multivariable models adjusted for sex, age, BMI, diabetes mellitus, hypertension, and smoking (Table 2). The unadjusted associations of plasma concentrations of choline and betaine with AF risk were almost log‐linear. These associations were, however, attenuated after multivariable adjustment (Figure). Additional adjustment for estimated glomerular filtration rate, fasting status, education level, serum folate, and plasma CRP did not appreciably alter the results (HR 1.10, 95% CI 1.00‐1.21, *P*=0.048 for choline; HR 1.16, 95% CI 1.04‐1.29, *P*=0.006 for betaine). Analyses on WECAC and NORVIT participants showed consistent results with HUSK, although in WECAC, plasma betaine was only marginally associated with AF risk after adjustments (Table 2). In WECAC, additional adjustment for fasting status made the associations slightly stronger (HR 1.17, 95% CI 1.05‐1.30, *P*=0.004 for choline; HR 1.12, 95% CI 1.01‐1.25, *P*=0.036 for betaine).

**Table 2:** Associations of Plasma Choline and Betaine With Incident AF Risk in HUSK, WECAC, and NORVIT^a^

Plasma Metabolite	Unadjusted		Adjusted for Sex and Age		Multivariable Model^b^	
	HR (95% CI)	*P* Value	HR (95% CI)	*P* Value	HR (95% CI)	*P* Value
HUSK (n=6949)						
Choline	1.45 (1.33‐1.57)	<0.001	1.12 (1.02‐1.22)	0.013	1.11 (1.02‐1.22)	0.015
Betaine	1.37 (1.26‐1.49)	<0.001	1.13 (1.02‐1.24)	0.014	1.18 (1.07‐1.29)	0.001
WECAC (n=3809)						
Choline	1.37 (1.24‐1.51)	<0.001	1.17 (1.05‐1.29)	0.004	1.14 (1.03‐1.27)	0.013
Betaine	1.16 (1.05‐1.28)	0.002	1.07 (0.96‐1.18)	0.22	1.10 (0.99‐1.22)	0.08
NORVIT (n=3733)						
Choline	1.30 (1.22‐1.39)	<0.001	1.17 (1.08‐1.26)	<0.001	1.15 (1.06‐1.24)	<0.001
Betaine	1.14 (1.06‐1.24)	0.001	1.14 (1.06‐1.24)	0.001	1.18 (1.09‐1.29)	<0.001
Pooled results^c^						
Choline	1.36 (1.30‐1.42)	<0.001	1.15 (1.09‐1.21)	<0.001	1.13 (1.08‐1.19)	<0.001
Betaine	1.22 (1.16‐1.29)	<0.001	1.12 (1.06‐1.18)	<0.001	1.16 (1.10‐1.22)	<0.001

AF indicates atrial fibrillation; BMI, body mass index; CI, confidence interval; HR, hazard ratio; HUSK, Hordaland Health Study; NORVIT, Norwegian B‐Vitamin Trial; WECAC, Western Norway Coronary Angiography Cohort.

a HRs (95% CIs) are reported per 1‐SD increment of natural log‐transformed plasma levels.

b Models were adjusted for sex, age (continuous), BMI (continuous), diabetes mellitus (yes/no), hypertension (yes/no), and smoking (yes/no).

c The results across the 3 cohorts were pooled using an inverse variance–weighted, fixed‐effect meta‐analysis.

**Figure 1:**
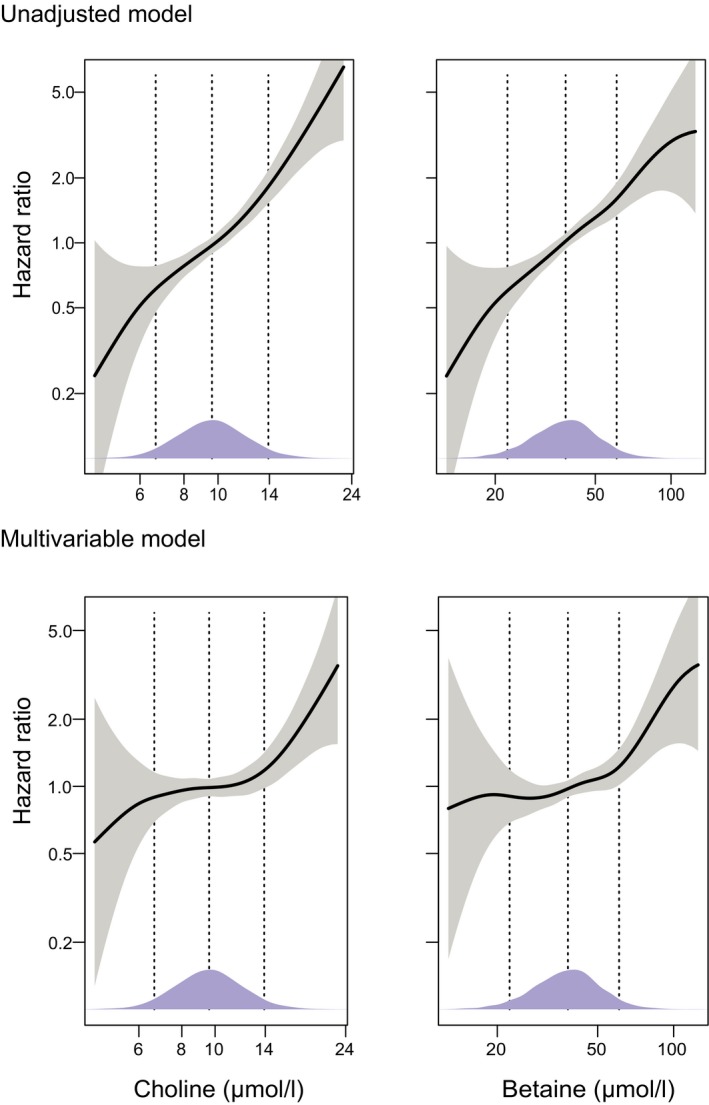
Associations of plasma choline and betaine with atrial fibrillation risk using generalized additive models in the Hordaland Health Study. Multivariable models were constructed with adjustment for sex, age, BMI (continuous), diabetes mellitus (yes/no), hypertension (yes/no), and smoking (yes/no). Both exposures were log‐transformed before entering the models. The solid lines show HRs, and the shaded areas 95% CIs. Density plots indicate the distribution of the biomarkers, and dotted lines denote the 5th, 50th, and 95th percentiles. BMI indicates body mass index; CI, confidence interval; HR, hazard ratio.

Moreover, additional adjustment for CVD at baseline gave similar associations in HUSK (HR 1.11, 95% CI 1.01‐1.21, *P*=0.025 for choline; HR 1.16, 95% CI 1.06‐1.28, *P*=0.002 for betaine). It resembled the results in WECAC after additional adjustment for coronary heart disease at baseline (HR 1.14, 95% CI 1.03‐1.27, *P*=0.014 for choline; HR 1.10, 95% CI 0.99‐1.22, *P*=0.09 for betaine).

In HUSK, the addition of plasma choline or betaine to the basic logistic regression model including sex, age, BMI, diabetes mellitus, hypertension, and smoking resulted in minor improvements in reclassification of participants at risk. The category‐free net reclassification index for the addition of choline and betaine in predicting AF was 0.02 (95% CI −0.07 to 0.11, *P*=0.646) and 0.14 (95% CI 0.06‐0.23, *P*=0.001), respectively. Similarly, the corresponding integrated discrimination index was 0.0014 (95% CI 0.000‐0.003, *P*=0.023) for choline and 0.003 (95% CI 0.001‐0.005, *P*=0.004) for betaine. In WECAC, the category‐free net reclassification index was 0.06 (95% CI −0.04 to 0.17, *P*=0.228) for choline and 0.06 (95% CI −0.05 to 0.16, *P*=0.286) for betaine, and the integrated discrimination index was 0.0019 (95% CI 0.000‐0.004, *P*=0.041) for choline and 0.001 (95% CI −0.001 to 0.003, *P*=0.219) for betaine. In NORVIT, the category‐free net reclassification index was 0.02 (95% CI −0.06 to 0.11, *P*=0.583) for choline and 0.14 (95% CI 0.06‐0.22, *P*=0.001) for betaine, and the integrated discrimination index was 0.0043 (95% CI 0.002‐0.007, *P*<0.001) for choline and 0.006 (95% CI 0.003‐0.009, *P*<0.001) for betaine.

### Associations Between Dietary Intakes of Choline and Betaine and AF Risk

Only weak correlations between dietary intakes and plasma concentrations were found for choline (Spearman ρ=0.06 in HUSK and −0.05 in WECAC) and betaine (Spearman ρ=0.12 in both HUSK and WECAC) (Table 3). Dietary intake of choline was marginally associated with AF risk (pooled HR 1.29, 95% CI 1.01‐1.66, fifth versus first quintile) after adjustment for sex, age, BMI, diabetes mellitus, hypertension, and smoking, whereas no significant association was observed between dietary betaine and AF risk (Table 4).

**Table 3:** Energy‐Adjusted Partial Correlation Coefficients^a^ Between Dietary Intakes and Plasma Concentrations of Choline and Betaine at Baseline in HUSK and WECAC

	Plasma Choline	Plasma Betaine
HUSK (n=5916)		
Total choline intake, mg/day	0.06^b^	−0.01
Betaine intake, mg/day	0.04^b^	0.12^b^
WECAC (n=1899)		
Total choline intake, mg/day	−0.05^b^	−0.02^b^
Betaine intake, mg/day	−0.05^b^	0.12^b^

HUSK, Hordaland Health Study; WECAC, Western Norway Coronary Angiography Cohort.

a Adjusted for sex and age (continuous).

b *P*<0.05.

**Table 4:** HRs (95% CIs) for AF Risk By Quintiles of Energy‐Adjusted Dietary Intakes of Choline and Betaine

	Quintiles of Dietary Intake					*P* Trend
	1 (Low)	2	3	4	5 (High)	
HUSK (n=5950)						
Total choline						
Unadjusted	1	1.15 (0.85‐1.56)	1.28 (0.95‐1.73)	1.29 (0.96‐1.74)	1.48 (1.11‐1.98)	0.006
Adjusted for sex and age	1	1.11 (0.82‐1.50)	1.17 (0.87‐1.57)	1.20 (0.89‐1.62)	1.43 (1.07‐1.92)	0.013
Multivariable model^a^	1	1.08 (0.80‐1.46)	1.13 (0.84‐1.52)	1.14 (0.85‐1.54)	1.32 (0.99‐1.78)	0.06
Betaine						
Unadjusted	1	0.95 (0.71‐1.27)	1.03 (0.78‐1.36)	1.03 (0.78‐1.36)	0.97 (0.73‐1.29)	0.99
Adjusted for sex and age	1	0.90 (0.68‐1.20)	1.02 (0.77‐1.35)	0.97 (0.74‐1.29)	0.95 (0.72‐1.27)	0.95
Multivariable model^a^	1	0.94 (0.71‐1.25)	1.06 (0.80‐1.40)	1.00 (0.75‐1.33)	0.99 (0.74‐1.32)	0.90
WECAC (n=1899)						
Total choline						
Unadjusted	1	1.18 (0.76‐1.85)	0.93 (0.58‐1.50)	1.45 (0.94‐2.24)	1.20 (0.76‐1.88)	0.26
Adjusted for sex and age	1	1.13 (0.72‐1.77)	0.94 (0.58‐1.51)	1.38 (0.89‐2.12)	1.31 (0.84‐2.07)	0.13
Multivariable model^a^	1	1.12 (0.72‐1.77)	0.92 (0.57‐1.48)	1.34 (0.87‐2.07)	1.23 (0.78‐1.93)	0.24
Betaine						
Unadjusted	1	1.76 (1.10‐2.82)	1.55 (0.96‐2.52)	1.57 (0.97‐2.54)	1.47 (0.91‐2.40)	0.32
Adjusted for sex and age	1	1.65 (1.03‐2.65)	1.60 (0.98‐2.59)	1.49 (0.92‐2.42)	1.39 (0.85‐2.25)	0.43
Multivariable model^a^	1	1.73 (1.08‐2.77)	1.67 (1.03‐2.71)	1.54 (0.95‐2.51)	1.47 (0.90‐2.41)	0.32
Pooled results^b^						
Total choline						
Unadjusted	1	1.16 (0.90‐1.49)	1.17 (0.91‐1.51)	1.34 (1.05‐1.71)	1.39 (1.09‐1.78)	0.004
Adjusted for sex and age	1	1.11 (0.86‐1.43)	1.10 (0.85‐1.41)	1.26 (0.98‐1.61)	1.40 (1.10‐1.78)	0.004
Multivariable model^a^	1	1.09 (0.85‐1.41)	1.06 (0.82‐1.37)	1.20 (0.94‐1.54)	1.29 (1.01‐1.66)	0.027
Betaine						
Unadjusted	1	1.12 (0.88‐1.44)	1.14 (0.90‐1.46)	1.14 (0.90‐1.46)	1.08 (0.84‐1.38)	0.58
Adjusted for sex and age	1	1.06 (0.83‐1.36)	1.14 (0.89‐1.45)	1.08 (0.85‐1.38)	1.05 (0.82‐1.34)	0.71
Multivariable model^a^	1	1.11 (0.87‐1.42)	1.18 (0.93‐1.51)	1.12 (0.87‐1.42)	1.10 (0.85‐1.41)	0.51

AF indicates atrial fibrillation; BMI, body mass index; CI, confidence interval; HR, hazard ratio; HUSK, Hordaland Health Study; WECAC, Western Norway Coronary Angiography Cohort.

a Models were adjusted for sex, age (continuous), BMI (continuous), diabetes mellitus (yes/no), hypertension (yes/no), and smoking (yes/no).

b The results across the 2 cohorts were pooled using an inverse variance–weighted, fixed‐effect meta‐analysis.

## Discussion

### Principal Findings

In our study involving 3 prospective cohorts, higher plasma concentrations of choline and betaine were associated with an increased AF risk. The risk associations were independent of traditional AF risk factors including older age, male sex, higher BMI, hypertension, diabetes mellitus, and smoking. In contrast, dietary intake of total choline was marginally associated with AF risk, and betaine intake was not associated.

### Previous Studies on Choline Metabolites and CVD Risk

Several studies have previously reported on the associations between systemic choline metabolites and CVD risk. A cross‐sectional analysis in HUSK suggested that high choline and low betaine plasma concentrations were associated with an unfavorable cardiovascular risk profile.^31^ A study among patients with established coronary heart disease reported that both high and low plasma betaine concentrations were associated with an increased risk of later coronary events and hospitalization for heart failure.^32^ In WENBIT patients with stable angina pectoris, elevated plasma choline levels were associated with an increased risk of acute myocardial infarction in nonsmoking patients.^6^

Regarding AF specifically, elevated plasma total homocysteine, a metabolite closely related to the choline oxidation pathway, has been associated with increased risk of AF recurrence following electrical cardioversion.^33^ In the Framingham Heart Study,^12^ no significant association between plasma betaine and risk of incident AF was reported, with age‐ and sex‐adjusted HR (95% CI) 1.03 (0.87‐1.22). In contrast, the corresponding pooled HR (95% CI) was 1.12 (1.06‐1.18) in our study. Several reasons may explain the discrepancy between the 2 findings. First, as the authors in the Framingham Heart Study stated, use of a strict threshold for corrected *P* values and limited power due to relatively small numbers of incident AF cases and participants may have masked moderate associations, probably including the betaine‐AF association. Second, the ascertainment of AF cases was heterogeneous. AF cases were ascertained by participant records in the Framingham Heart Study but by hospital discharge codes in our study, which possibly leads to different degrees of underestimation of new‐onset AF in the 2 studies. Third, the Framingham Heart Study used fasting plasma samples, whereas we used mostly nonfasting samples, which may somewhat contribute to the inconsistency.

### Possible Mechanisms

The mechanisms behind the relationship of dietary choline and betaine with CVD risk remain elusive. One study from the United States suggested an inverse association between choline intake and risk of stroke, a major adverse event related to AF.^34^ However, a recent large meta‐analysis of observational data concluded that there was no relationship between dietary choline and betaine and CVD risk.^35^ Accordingly, in our study, the correlations between dietary and systemic choline and betaine were weak or absent, in line with previous studies.^36^ Moreover, we found a weak association of total choline intake, but no association of betaine intake, with incident AF. The positive relationships between systemic choline and betaine and AF risk are therefore more likely related to metabolic rather than to dietary factors.

First, the choline metabolic pathway is partially taking place inside the mitochondrion, and it is therefore interesting that experimental data in rodents suggest a causal link between increased mitochondrial oxidative stress and AF.^37^ Oxidative stress may impair betaine‐homocysteine methyltransferase, resulting in accumulation of circulating choline and betaine.^38^ Second, high plasma concentrations of choline and betaine have been associated with elevated concentrations of trimethylamine N‐oxide.^39^ This metabolite is related to microbiomic processing of dietary choline and to a lesser extent betaine, and systemic trimethylamine N‐oxide has been positively associated with adverse cardiovascular events.^40^ Third, systemic choline and betaine were shown to be related to lipid metabolism.^41^ Higher plasma choline may downregulate apolipoprotein A1, the major protein of high‐density lipoprotein cholesterol, which may consequently facilitate AF.^42^ However, associations between lipid‐related genetic variants and AF risk have recently been challenged.^43^ Moreover, the association between betaine and AF risk was less strong in patients with suspected stable angina pectoris than in the general population. This may due to complex interactions of choline oxidation with extensive use of medications among the WECAC patients.^44^ Fourth, intravascular volume status may alter the susceptibility to AF.^45^ Notably, betaine is an important osmolyte, ie, a regulator of cellular volume,^4^ suggesting that increased plasma levels could be associated with AF due to altered cellular osmotic stress. Fifth, the choline metabolism is an important determinant of methylation status in the liver, especially the flux over the betaine‐homocysteine methyltransferase enzyme.^46^ Several studies have suggested that AF is associated with methylation of genetic loci related to AF susceptibility.^47^, ^48^ Although any relationship between choline metabolism and methylation specifically in the myocardium is elusive, it is possible that the present findings reflect methylation status and epigenetic regulation relevant to AF development and persistence.

### Strengths and Limitations

To the best of our knowledge, this is the first study examining associations of plasma and dietary choline and betaine with subsequent AF risk. The major strengths of this study include the large number of participants recruited from 3 independent cohorts with different health profiles, measurements of exposures both as plasma concentrations and dietary intakes, along with long‐term prospective follow‐up. However, the study has several limitations that merit attention. The ascertainment of AF was based on hospitalization data only, which implies that cases with asymptomatic AF without hospitalization might not have been included. In NORVIT, we had no baseline information on any previous AF, making it impossible to state whether a later diagnosis of AF was truly an incident event. However, the risk estimates in NORVIT were in general similar to those observed in HUSK and WECAC, making it less likely that the choline and betaine AF risk associations in NORVIT merely reflected persistent or recurrent AF present at baseline. Moreover, the within‐person reproducibility for neither plasma betaine nor choline is excellent.^44^, ^49^ Therefore, the risk associations explored based on baseline samples may be underestimated due to regression dilution bias.^50^

## Conclusions

The current study indicates that higher plasma concentrations of choline and betaine are associated with an increased risk of subsequent AF, independent of traditional AF risk factors. Moreover, dietary intake of choline, but not betaine, is associated with the risk of AF. This suggests alterations in choline metabolism being implicated in AF pathogenesis.

## Disclosures

None.
